# Genetic Association of Phosphodiesterases With Human Cognitive Performance

**DOI:** 10.3389/fnmol.2019.00022

**Published:** 2019-02-08

**Authors:** Mark E. Gurney

**Affiliations:** Tetra Discovery Partners, Grand Rapids, MI, United States

**Keywords:** phosphodiesterase, PDE1C, PDE2A, PDE4B, PDE4D, BDNF, cognition, brain derived neurotrophic factor

## Abstract

Recent, large-scale, genome-wide association studies (GWAS) provide a first view of the genetic fine structure of cognitive performance in healthy individuals. These studies have pooled data from up to 1.1 million subjects based on simple measures of cognitive performance including educational attainment, self-reported math ability, highest math class taken, and pooled, normalized scores from cognitive tests. These studies now allow the genome-wide interrogation of genes and pathways for their potential impact on human cognitive performance. The phosphodiesterase (PDE) enzymes regulate key cyclic nucleotide signaling pathways. Many are expressed in the brain and have been the targets of CNS drug discovery. Genetic variation in PDE1C, PDE4B and PDE4D associates with multiple measures of human cognitive function. The large size of the human PDE4B and PDE4D genes allows genetic fine structure mapping to transcripts encoding dimeric (long) forms of the enzymes. Upstream and downstream effectors of the cAMP pathway modulated by PDE4D [adenylate cyclase 1 (ADCY1), ADCY8, PRKAR1A, CREB1, or CREBBP] did not show genetic association with cognitive performance, however, genetic association was seen with brain derived neurotrophic factor (BDNF), a gene whose expression is modulated by cAMP. Notably absent was genetic association in healthy subjects to targets of CNS drug discovery designed to improve cognition in disease states by the modulation of cholinergic [acetylcholinesterase (ACHE), choline acetyltransferase (CHAT), nicotinic alpha 7 acetylcholine receptor (CHRNA7)], serotonergic (HTR6), histaminergic (HRH3), or glutamatergic (GRM5) pathways. These new data provide a rationale for exploring the therapeutic benefit of selective inhibitors of PDE1C, PDE4B and PDE4D in CNS disorders affecting cognition.

## Introduction

Multiple recent, large-scale, genome-wide association studies (GWAS) provide an unbiased view of the genetic fine structure of cognitive function in healthy individuals (Lam et al., 2017; Davies et al., 2018; Lee et al., 2018; Savage et al., 2018). The studies ranged in size from 107,207 to 1.1 million individuals. The collection of phenotypic information across such a large number of subjects required the use of very simple phenotypes of cognitive ability. In the largest GWAS study, which was conducted by the Social Science Genetic Association Consortium (SSGAC) and 23andMe, educational attainment was supplemented by analyses of self-reported math ability, highest math class, and by a composite score for cognitive performance that allowed the pooling of data collected from subjects across multiple studies that utilized different cognitive assessment tools (Lee et al., 2018). The study was limited to subjects of European ancestry and had lower predictive power in African-Americans. Educational attainment more broadly captures cognitive ability, but also personality and motivation, as well as behavior traits such as orderliness, lack of impulsiveness, and perseverance. The conclusions of the study are subject to the concerns that educational attainment is strongly influenced by socioeconomic status, by parental education level, nutrition, early childhood experience, the quality of schooling, and other environmental factors. For example, household income explains 7% of the population variation in educational attainment in the GWAS cohort (Lee et al., 2018). Remarkably, genetic variation was found to account for up to 11% of the population variation in educational attainment.

The genetic contribution to educational attainment comes from many genes of small effect. The GWAS reported by SSGAC and 23andMe identified 1,271 single nucleotide polymorphisms (SNPs) that reached genome-wide significance. The genes identified were overwhelmingly expressed in neurons. Genes expressed principally in glial cells were largely absent (Lee et al., 2018). The GWAS thus provides a rich resource for testing the contribution of candidate genes to human cognition. This allows the interrogation of gene families and pathways for potential targets of therapeutic intervention.

## Genetic Association of Phosphodiesterases (PDE) With Educational Attainment and Cognitive Performance

The phosphodiesterase (PDE) enzymes hydrolyze cyclic AMP or cyclic GMP, key intracellular signaling molecules, to the respective monophosphate (AMP or GMP). They comprise a large gene family whose protein products can be modulated by small molecule therapeutics (Bender and Beavo, 2006; Conti and Beavo, 2007). Cilostazol, a small molecule inhibitor of PDE3 is used to treat intermittent claudication in peripheral arterial occlusive disorder; sildenafil, tadalafil, and vardenafil, inhibitors of PDE5, are used to treat male erectile dysfunction; and roflumilast and apremilast, which inhibit all four subtypes of PDE4, are used to treat chronic obstructive pulmonary disorder, psoriasis or psoriatic arthritis (Yuan et al., 2013; Bedenis et al., 2014; Deeks, 2015; Garnock-Jones, 2015). Many of the PDE gene family members are expressed in brain and have been utilized as targets for CNS drug discovery. PDE4, PDE9 and PDE10 inhibitors have been explored in major depression, Alzheimer’s disease and schizophrenia, although clinical data are largely negative (Zhu et al., 2001; Wu et al., 2018).

**Figure 1:**
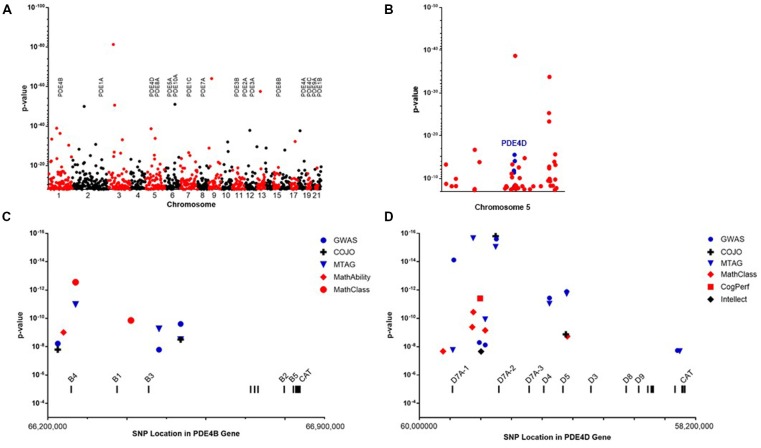
**(A)** Manhattan plot showing the location and *P*-values for single nucleotide polymorphisms (SNPs) reaching genome-wide significance in the primary genome-wide association genome-wide association study (GWAS) of educational attainment (Lee et al., 2018). The locations of genes encoding phosphodiesterase (PDE) are shown. **(B)** SNP reaching genome-wide significance on chromosome 5. Informative SNP covering the PDE4D gene are colored blue. **(C)** Genetic fine structure of the PDE4B gene. SNP achieving genome-wide significance in the GWAS (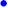), conditional-joint analysis (COJO; **+**) or multi-trait analysis of GWAS (MTAG; 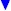) analyses cluster at the 5’end of the gene. These overlap SNP associating with self-reported math ability (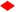) and highest math class taken (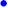). The 5’ exons of mRNA encoding the major PDE4B isoforms are labeled. A group of 3’ exons encoding the catalytic domain are common to all PDE4B mRNA. **(D)** Genetic fine structure of the PDE4D gene. SNP achieving genome-wide significance in the GWAS (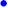), COJO (**+**) or MTAG (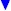) analyses cluster at the 5’end of the gene. These overlap SNP associating with self-reported math ability(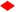), cognitive performance (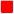) and SNP associating with intellect (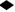) in an independent study by Savage et al. (2018). Genome position is based on the GRCh37.p13: Annotation Release 105.

The large GWAS dataset from the SSGCA and 23andMe study provides a means to test the contribution of genetic variation in PDE gene family members to cognitive performance (Figure 1). SNP in PDE1A, PDE1C, PDE2A, PDE4B and PDE4D reached genome-wide significance (*P* < 5 × 10^−8^) in the primary GWAS which used educational attainment as a phenotype (Table 1). Several informative SNP in PDE1A (rs1835339) and PDE4B (rs556755587, rs11208742) were located immediately upstream of the respective genes, while the remaining SNP were intragenic.

**Table 1:** Genetic association results for phosphodiesterase (PDE) genes.

Gene	Chr	Start	End	SNP	Position	Location	GWAS-Eduyears	COJO-Eduyears	MTAG-eduyears	Math ability	Highest math class	Cognitive performance
PDE1A	2	183,004,762	183,387,572	rs1835339	183,393,680	upstream	5.590E-09					
PDE1A	2	183,004,762	183,387,572	rs4666851	183,378,875	intragenic			4.470E-11			
PDE1B	12	54,943,177	54,973,023	null								
PDE1C	7	31,791,666	32,339,016	rs9771228	32,322,496	intragenic	5.910E-11	5.977E-11	9.187E-13			
PDE1C	7	31,791,666	32,339,016	rs4720058	32,291,774	intragenic						2.192E-12
PDE1C	7	31,791,666	32,339,016	rs7798739	32,292,961	intragenic				1.114E-08	1.786E-10	
PDE2A	11	72,287,184	72,385,497	rs72962169	72,365,669	intragenic	1.260E-19	1.914E-19	5.239E-21			
PDE3A	12	20,522,179	20,840,575	null								
PDE3B	11	14,665,191	14,893,605	null								
PDE4A	19	10,527,449	10,580,307	null								
PDE4B	1	66,258,193	66,840,262	rs55675587	66,224,881	upstream	5.920E-09	1.580E-08				
PDE4B	1	66,258,193	66,840,262	rs11208742	66,239,487	upstream				9.634E-10		
PDE4B	1	66,258,193	66,840,262	rs11208757	66,269,936	intragenic			1.038E-11		2.753E-13	
PDE4B	1	66,258,193	66,840,262	rs11208774	66,410,109	intragenic	1.610E-08		5.433E-10		1.383E-10	
PDE4B	1	66,258,193	66,840,262	rs1392816	66,481,188	intragenic	2.440E-10	3.056E-09				
PDE4B	1	66,258,193	66,840,262	rs72667460	66,536,012	intragenic			3.156E-09			
PDE4C	19	18,318,771	18,359,010	null								
PDE4D	5	58,264,865	59,783,925	rs13361043	58,318,963	intragenic	1.840E-08					
PDE4D	5	58,264,865	59,783,925	rs61511922	58,304,269	intragenic	1.280E-12		2.056E-08			
PDE4D	5	58,264,865	59,783,925	rs7736817	59,036,578	intragenic		1.567E-16			1.841E-09	
PDE4D	5	58,264,865	59,783,925	rs981230	59,039,858	intragenic	3.630E-12		1.892E-12			
PDE4D	5	58,264,865	59,783,925	rs1960603	59,045,193	intragenic	2.500E-16					
PDE4D	5	58,264,865	59,783,925	rs79798166	59,152,140	intragenic		1.331E-09	9.166E-12			
PDE4D	5	58,264,865	59,783,925	rs2910823	59,498,175	intragenic	7.550E-09					
PDE4D	5	58,264,865	59,783,925	rs966221	59,502,520	intragenic	5.020E-09		8.896E-16			
PDE4D	5	58,264,865	59,783,925	rs4283754	59,570,258	intragenic			1.155E-10		6.966E-10	
PDE4D	5	58,264,865	59,783,925	rs7735958	59,603,211	intragenic						3.895E-12
PDE4D	5	58,264,865	59,783,925	rs13154429	59,608,950	intragenic	7.520E-15					
PDE4D	5	58,264,865	59,783,925	rs35335033	59,647,992	intragenic					3.617E-11	
PDE4D	5	58,264,865	59,783,925	rs7737905	59,648,716	intragenic			2.173E-16			
PDE4D	5	58,264,865	59,783,925	rs4699955	59,654,979	intragenic					4.103E-10	
PDE4D	5	58,264,865	59,783,925	rs27220	59,775,136	intragenic	7.520E-15					
PDE4D	5	58,264,865	59,783,925	rs11746901	59,781,702	intragenic			1.708E-08			
PDE4D	5	58,264,865	59,783,925	rs72755130	59,844,983	upstream					2.109E-08	
PDE5A	4	120,415,550	120,549,981	null								
PDE7A	8	66,626,569	66,753,969	null								
PDE8A	5	76,476,082	76,724,081	null								
PDE8B	15	85,523,744	85,682,376	null								
PDE9A	21	44,073,862	44,195,619	null								
PDE10A	6	165,740,776	166,075,588	null								

*Association results are tabulated for the independent single nucleotide polymorphism (SNP) that reached genome-wide significance (*P* < 5 × 10^−8^) in the pooled-sex cohort. The genome position is based on GRCh37.p13: Annotation Release 105. *P*-values are shown in the table, as in Figure 1; null indicates that no SNP reached genome-wide significance in the GWAS. The PDE6 family of photoreceptor PDEs was excluded from the analysis (Bender and Beavo, 2006). Abbreviations: GWAS, genome-wide association analysis; EduYears, Educational attainment as measured by highest level of education achieved; COJO, Conjoint analysis; MTAG, Multi-Trait Analysis of GWAS*.

The SSGCA and 23andMe study also subjected the GWAS dataset to a more stringent, conditional-joint analysis (COJO) (Yang et al., 2012). The genetic association of PDE1A was lost, while SNP covering PDE1C, PDE2A, PDE4B and PDE4D continued to achieve genome-wide significance (Table 1). The SNP data also were analyzed using multi-trait analysis of GWAS (MTAG) which increases the number of SNP identified at genome-wide significance (Turley et al., 2018). The MTAG analysis focused on three complementary cognitive phenotypes: self-reported math ability, highest math class taken, and cognitive performance. The two math phenotypes were self-reported by participants in 23andMe. The measure of cognitive performance was constructed through a meta-analysis of published results from the COGENT consortium (Trampush et al., 2017). MTAG confirms the genetic association with educational years for PDE1A, PDE1C, PDE2A, PDE4B and PDE4D (Table 1). PDE1C also shows genetic association by MTAG across all of the cognitive subanalyses. Multiple SNP over PDE4B and PDE4D reach genome-wide significance for highest math class taken, while only a single SNP upstream of PDE4B continues to reach genome-wide significance for self-reported math ability (rs11208742; *P* = 9.6 × 10^−10^) and a single SNP in PDE4D reaches genome-wide significance for cognitive performance (rs7735959; *P* = 3.89 × 10^−12^). As a credibility check, the MTAG for cognitive performance was repeated with the COGENT cohort excluded. SNP in PDE1C and PDE4D continue to reach genome-wide significance. The complete data listings extracted from the SSGCA and 23andMe study are presented in Supplementary Tables S1–S6.

Notably absent from the GWAS are genes previously pursued as targets for cognitive enhancing drugs. Absent are components of the cholinergic neurotransmitter pathway [acetylcholinesterase (ACHE), choline acetyltransferase (CHAT), nicotinic alpha 7 acetylcholine receptor (CHRNA7)], the serotonergic pathway (HT6R, serotonin receptor 6), or the histaminergic (HRH3, histamine receptor 3) and glutamatergic pathways (GRM5 -metabotropic glutamate receptor 5). Nor do components of the amyloid pathway [amyloid protein precursor (APP); apolipoprotein E (APOE)] or components of the complement pathway linked to synaptic pruning by microglial cells (C4A, complement 4A; C4B, complement 4B) show genetic association with cognition in the GWAS data set (Supplementary Table S7).

The genes for PDE4B and PDE4D have dense SNP coverage due to their large size. On average, human genes are 67,000 base pairs (bps) in length (Piovesan et al., 2016). GWAS studies typically are conducted with methods that allow the scanning of 1 million or more SNP per genome. These on average are located every 1,000–3,000 bps across the 3.1 billion bps of the human genome. Thus, a typical gene is covered by 20–30 SNP in a GWAS. Adjacent SNP identify blocks of DNA that are in linkage disequilibrium (LD). The large size of the genes for PDE4B (582,069 bp) and PDE4D (1,519,060 bp) provides proportionally denser coverage in a GWAS. Given that each of these genes utilize multiple promoters and complex splicing to generate multiple protein isoforms, (Gretarsdottir et al., 2003; Fatemi et al., 2008; Guan et al., 2012) the density of SNP coverage provides insight into the genetic fine structure of the PDE4B and PDE4D genes (Figure 1).

## PDE4B

**Figure 2:**
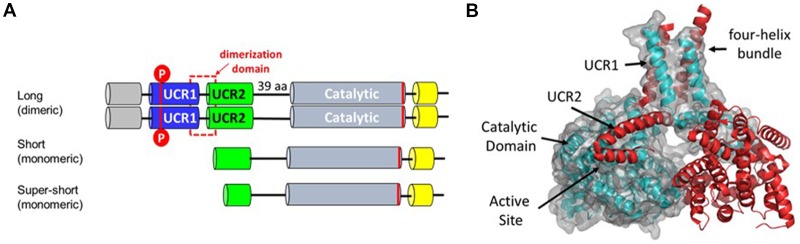
**(A)** PDE4 protein isoforms. Each of the PDE4 genes produces multiple transcripts that vary in 5’ exon structure (Bender and Beavo, 2006). Dimeric forms of PDE4 enzymes are encoded by long transcripts that vary in 5’ exons but all contain 3’ exons encoding the dimerization, linker and catalytic domains. Short and super-short transcripts encoding monomeric isoforms lack exons encoding the dimerization domain. Basal PDE4 enzymatic activity is increased by PKA phosphorylation of UCR1 (Sette and Conti, 1996; Hoffmann et al., 1998). **(B)** Protein structure of the PDE4 dimer based on PDB ID:4WZI (Cedervall et al., 2015). The dimerization domain is formed by a four helix bundle comprising the last alpha-helix in UCR1 and the first alpha-helix in UCR2 (Cedervall et al., 2015; Richter and Conti, 2002). A regulatory helix from UCR2 is positioned in *trans*-across the active site of the opposite monomer and thereby regulates hydrolytic activity by controlling access to cAMP (Burgin et al., 2010).

PDE4 genes encode multiple protein isoforms that are present as either dimers or monomers within the cell (Figure 2). Informative SNP in the gene encoding PDE4B cluster at the 5’ end of the gene across exons that through differential promoter utilization and splicing encode dimeric forms of the enzyme known as PDE4B4, B1 and B3 with each transcript beginning with a unique, 5’ exon (Bender and Beavo, 2006). The PDE4B gene also encodes transcripts for monomeric forms of PDE4B known as PDE4B2 and PDE4B5 (Campbell et al., 2017). These transcripts utilize a downstream promoter and lack the upstream exons needed to encode the protein domains necessary for dimerization (Richter and Conti, 2002; Cedervall et al., 2015). The absence of genetic association with 3’ SNP indicates that the dimeric forms as compared to the monomeric forms of PDE4B have greater effect on human cognition. Activity of the PDE4B dimer is increased by protein kinase A phosphorylation, (Houslay and Adams, 2003) whereas levels of the monomeric PDE4B isoforms are controlled transcriptionally (Jin and Conti, 2002). Transcriptional up-regulation of PDE4B2 in monocytes and microglia allows production of Tumor Necrosis Factor-α (TNF α) by inflammatory cells in response to Toll receptor-4 pathway activation (e.g., by bacterial lipopolysaccharide or LPS; Jin and Conti, 2002; Zhang et al., 2002; Jin et al., 2005; Wilson et al., 2017).

PDE4B gene variants previously were linked to risk for schizophrenia in a study of 878 schizophrenic or schizoaffective patients and 604 controls that included both Caucasian and African American subjects. In contrast to SNP associated with cognition, SNP associated with schizophrenia clustered at the 3’ end of the PDE4B gene in a block of LD extending from 66,750,560 to 66,829,764 bp on Chr 1. Genetic association of the 3’ end of the PDE4B gene with schizophrenia was replicated in Japanese and Han Chinese (Numata et al., 2008; Guan et al., 2012). This LD blocks contains exons encoding transcripts for the PDE4B2 and PDE4B5 monomeric forms of PDE4B. If variant monomeric forms of PDE4B are causal in schizophrenia, their expression in brain microglia could be relevant to the etiology of the disease though an effect on synaptic pruning (Sekar et al., 2016).

Dimeric forms of PDE4B interact with a binding partner known as disrupted in schizophrenia 1 (DISC1), which holds the enzyme in a closed, inactive conformation (Millar et al., 2005). DISC1 was identified in a Scottish kindred in association with psychiatric disorders that also affect cognition (e.g., schizophrenia, bipolar disorder and major depression; Millar et al., 2000; Blackwood and Muir, 2004). However, no SNP covering DISC1 reach genome-wide significance in the GWAS data set.

## PDE4D

The much larger PDE4D gene is covered by 16 informative SNP that are all intragenic (Figures 1, 2). These cluster over the 5’ end of the gene and four of the seven SNP cover the first three exons of the transcript for a dimeric form of PDE4D known as PDE4D7 (Gretarsdottir et al., 2003; Bender and Beavo, 2006). Two additional SNP cover the 5’ end exons for transcripts encoding the PDE4D4 and PDE4D5 dimeric forms of the enzyme. PDE4D7, D4 and D5 are expressed in brain and their enzymatic activity is increased by PKA phosphorylation (Richter et al., 2005).

An independent GWAS in 269,867 individuals of European ancestry using a meta-analysis of scores on neurocognitive tests independently confirmed association of gene variants in PDE4D with cognitive performance (Savage et al., 2018). The analysis identified independent significant SNP and then grouped these under a lead SNP to identify regions in approximate linkage equilibrium with each other. For PDE4D, the lead SNP was identified as rs34426618. This identified an LD block on chromosome 5 (bps 59,572,804–59,666,806) at the 5’ end of the PDE4D gene between the first two exons of the PDE4D7 transcript (D7A1 and D7A2) with genome-wide significance for cognition (*P* = 2.16 × 10^−8^). SNP over other PDE were not associated with neurocognitive performance. Davies et al. (2018) in a third GWAS of 300,486 also identified a 5’ SNP over PDE4D (rs36004779) at genome-wide significance. Lam et al. (2017) previously reported genome-wide significance for PDE4D in a GWAS incorporating 107,207 individuals of European ancestry. Xu et al. (2017) further reported suggestive association of PDE4D with cognition in a Chinese study of adult twins. Thus, the 5’end of the PDE4D gene that encodes dimeric isoforms of the enzyme is a consistent hit across multiple GWAS using varying measures of neurocognitive performance.

Genetic association of PDE4D with stroke was shown previously in an Icelandic GWAS study (Gretarsdottir et al., 2003). As in the SSGCA and 23andMe study, informative SNP were located over 5’ exons encoding dimeric forms of PDE4D including three LD blocks over the 5’ exons encoding the dimeric PDE4D7 isoform. The Icelandic study identified both at-risk and protective haplotypes and showed that the at-risk haplotype altered expression of particular PDE4D transcripts such as PDE4D7. Thus, genetic association of 5’ exons of PDE4D with educational attainment and cognitive performance may also be due to subtle changes in expression of particular PDE4D isoforms.

Exome sequencing in children with a rare neurodevelopmental disorder identified PDE4D mutations as causative for acrodysostosis type 2 with or without hormone resistance (ACRDYS2; Lee et al., 2012; Linglart et al., 2012; Michot et al., 2012). ACRDYS2 causes severe intellectual disability accompanied by brachydactyly (short fingers and toes) and nasal hypoplasia. These ultra-rare mutations in PDE4D arise *de novo* during gametogenesis. The ACRDYS2 mutations are autosomal dominant, and thus of large effect, and are exclusively missense mutations. These mutations contrast with the PDE4D gene variants in the GWAS as these are common variants of small effect. As do the SNP showing genetic association in the GWAS scan, the ACRDYS2 mutations implicate the PDE4D dimer in normal brain function. Mutations alter amino acid residues needed for PDE4D dimerization and affect regulation of enzymatic activity by PKA phosphorylation (Cedervall et al., 2015; Gurney et al., 2015; Motte et al., 2017).

The high sequence conservation of PDE4D is noteworthy. PDE4D7, a representative dimeric form of PDE4D, is highly conserved across species with >99% amino acid sequence conservation across the 748 amino acid length of the polypeptide. The core amino acid sequence of PDE4D7 from UCR1 through the end of the catalytic domain is absolutely conserved across all species except for a phenylalanine in UCR2 that is unique to primate (phenylalanine271) which in non-primate species is a tyrosine (Burgin et al., 2010). That single amino acid difference may be an important biological adaption in primates as phenylalanine271 projects from UCR2 into the catalytic site. Modeling studies suggest that in non-primate species, the tyrosine hydroxyl forms a hydrogen bond with the cAMP ribose (Burgin et al., 2010). This affects K_M_ as shown be reciprocal exchange of the residues between PDE4D and PDE4B with the phenylalanine lowering K_M_ from 6 μM to 1 μM. Thus, primate PDE4D may function within cells at lower cAMP concentration as compared to the non-primate enzyme. This could be relevant to adaptions in primates that enhance dendritic compartmentalization in cortical neurons (Beaulieu-Laroche et al., 2018).

## PDE4D-PKA-CREB Pathway Analysis

The role of PDE4 in learning and memory has been studied extensively in the *Drosophila* fruit fly and in mice. Mutational studies in fruit flies identified PDE4 as a key modulator of a signaling pathway critical for associative learning, courtship behavior and neurodevelopment (Byers et al., 1981). Deletions of the single PDE4 gene in *Drosophila*, known as *dnc* -dunce, disrupt learning and memory by preventing the hydrolysis of cAMP and thereby altering the normal spatial and temporal patterning of cAMP signaling (Byers et al., 1981; Gervasi et al., 2010). This is a key aspect of cAMP signaling across multiple cells and tissues (Willoughby et al., 2006; Castro et al., 2010; Blackman et al., 2011). Mutations in a second gene, known as *rut*-rutabaga, identified calcium-calmodulin dependent adenylate cyclase (ADCY) as the upstream source of cAMP important for learning and memory (Levin et al., 1992). In mammals, two calcium-calmodulin dependent ADCYs (ADCY1 and ADCY8) link Ca^2+^ influx through the N-methyl D-aspartate (NMDA) receptor to early stages of memory formation (Wong et al., 1999). Further genetic analysis in mice and flies identified CREB -cAMP response element binding protein, as the key downstream effector (Milner et al., 1998). CREB acts with a second protein, CREBBP—CREB binding protein, to regulate gene transcription (Chrivia et al., 1993). Haplo-insufficiency in humans causes Rubinstein-Taybi Syndrome, a neurodevelopmental disorder that causes intellectual disability, while the homozygous gene deletions are lethal (Petrij et al., 1995; Tanaka et al., 2000). However, SNP over ADCY1, ADCY8, CREB1, and CREBBP do not reach genome-wide significance in the GWAS. This lack of genetic association with upstream and downstream effector molecules highlights the tight biochemical regulation of the key signaling molecule, cAMP, by a critical hydrolytic enzyme, PDE4D.

The gene for brain derived neurotrophic factor (BDNF), contains promoter elements known as cAMP response elements that are required for CREB-mediated gene expression (Tabuchi et al., 2002). Studies in mice indicate that PDE4D modulates expression of BDNF through phosphorylation of CREB (Zhang et al., 2018). SNP in BDNF reach genome-wide significance in the GWAS, COJO and MTAG for educational attainment and in the MTAG for highest math class (Table 2). BDNF is processed to the mature form by proteolysis of a pro-peptide (Leibrock et al., 1989). A single amino acid polymorphism in the pro-peptide, Val66Met is associated with major depression and response to anti-depressant treatment (Tsai, 2018). Two of the SNP identified in the GWAS map to the 5’ end of the BDNF (rs962369 and rs12273363) and therefore potentially mark an LD block containing the Val66Met variant. The BDNF Val66Met variant is associated with increased decline in cognitive performance in Alzheimer’s disease (Gomar et al., 2016; Boots et al., 2017). The GWAS implies that genetic variation in BDNF may impact more broadly on cognition in healthy individuals.

**Table 2:** Association Results for brain derived neurotrophic factor (BDNF).

Analysis	Chr	Start	End	SNP	Position	*P*-value
GWAS	11	27,676,440	27,743,605	rs11030102	27,681,596	4.600E-10
COJO	11	27,676,440	27,743,605	rs12273363	27,744,859	7.3750E-04
MTAG	11	27,676,440	27,743,605	rs138385919	27,690,566	2.867E-11
MTAG	11	27,676,440	27,743,605	rs138385919	27,690,566	2.867E-11
HighestMathClass	11	27,676,440	27,743,605	rs962369	27,734,420	3.581E-11

*The Informative SNP, the genome position of the SNP, and P-value for each analysis are shown*.

## Conclusions

The SSGCA and 23andMe GWAS study of educational attainment and cognitive performance identified 1,271 independent SNP at genome-wide significance which cover 10% of the 20,000 or so genes encoded by the human genome. The vast majority of these SNP occur in intragenic regions of uncertain function. Recombination events during individual meiosis to create a sperm or an egg are relatively rare. Thus, adjacent regions of DNA tend to be transmitted as blocks from parents to child. This causes adjacent genetic markers on a single chromosome to be in LD due to their lower probability of being separated by recombination during meiosis. Across a population, meiotic events are pooled in a GWAS study, such that the blocks of DNA in LD become smaller. This has two effects in a GWAS study, genome-wide significance may be “inflated” by genetic markers that are in LD, and more importantly, the informative SNP may not be the cause of the biological variation, but instead mark a block of DNA that contains the key variant.

This methodology was good enough to reflect known biology as shown by an analysis of the PDE gene family. PDE1A, PDE1C, PDE2, PDE4B and PDE4D have plausible links to cognitive function. Selective inhibitors improve cognitive performance in diverse animal models although human clinical data are not yet available (Heckman et al., 2015). Further genetic data link PDE4B and PDE4D to human cognition. Genetic variation in PDE4B has been linked to schizophrenia, while ACRYDS2 mutations in PDE4D severely affect cognitive function. (Fatemi et al., 2008; Lee et al., 2012; Linglart et al., 2012; Michot et al., 2012). As shown in the Icelandic study, SNP in the PDE4D gene thought to be associated with stroke had subtle effects on PDE4D expression and even on the expression of transcripts encoding different PDE4D isoforms (Gretarsdottir et al., 2003). Thus, the PDE enzymes provide a rich source of targets for drug discovery and development. The recent large scale GWAS studies of human cognitive ability suggest the potential of PDE1C, PDE4B and PDE4D inhibitors to address cognitive impairment across multiple CNS disorders.

## Author Contributions

MG wrote the manuscript.

## Conflict of Interest Statement

MG is an employee of Tetra Discovery Partners, Inc. which has a financial interest in the discovery and development of PDE4B and PDE4D allosteric inhibitors for the treatment of Fragile X Syndrome, Alzheimer’s disease and other CNS disorders.
